# Incidence and Mortality of Solid Cancers in People Exposed *In Utero* to Ionizing Radiation: Pooled Analyses of Two Cohorts from the Southern Urals, Russia

**DOI:** 10.1371/journal.pone.0160372

**Published:** 2016-08-03

**Authors:** Alexander Akleyev, Isabelle Deltour, Lyudmila Krestinina, Mikhail Sokolnikov, Yulia Tsareva, Evgenia Tolstykh, Joachim Schüz

**Affiliations:** 1 Urals Research Center for Radiation Medicine, Chelyabinsk, Russian Federation; 2 Chelyabinsk State University, Chelyabinsk, Russian Federation; 3 Section of Environment and Radiation, International Agency for Research on Cancer (IARC), Lyon, France; 4 Southern Urals Biophysics Institute, Ozyorsk, Russian Federation; University of South Carolina, UNITED STATES

## Abstract

**Background:**

Previous studies have shown that acute external *in utero* exposure to ionizing radiation can increase cancer risk. It is not known whether chronic exposure at low dose rates, including due to radionuclide intake, influences the lifetime risk of solid cancers in the offspring. The objective of this study was to investigate solid cancer risk after *in utero* irradiation.

**Methods:**

Cancer incidence and mortality over a 60-year period (from January 1950 to December 2009) were analyzed in the Urals Prenatally Exposed Cohort (UPEC). The cohort comprised *in utero* exposed offspring of Mayak Production Association female workers and of female residents of Techa River villages. Some of the offspring also received postnatal exposure, either due to becoming radiation workers themselves or due to continuing to live in the contaminated areas of the Techa River. The mortality analyses comprised 16,821 subjects (601,372 person-years), and the incidence analyses comprised 15,813 subjects (554,411 person-years). Poisson regression was used to quantify the relative risk as a function of the *in utero* soft tissue dose (with cumulative doses up to 944.9 mGy, mean dose of 14.1 mGy in the pooled cohort) and the postnatal stomach dose for solid cancer incidence and mortality.

**Results:**

When a log-linear model was used, relative risk of cancer per 10 mGy of *in utero* dose was 0.99 (95% confidence interval (CI) = 0.96 to 1.01) based on incidence data and 0.98 (CI = 0.94 to 1.01) based on mortality data. Postnatal exposure to ionizing radiation was positively associated with the solid cancer risk in members of the UPEC, with a relative risk of 1.02 per 10mGy CI = 1.00 to 1.04).

**Conclusions:**

No strong evidence was found that chronic low-dose-rate exposure of the embryo and fetus increased the risk of solid cancers in childhood or in adulthood. For both incidence and mortality, a tendency towards a decreased relative risk was noted with increasing doses to soft tissues of the fetus. Further follow-up will provide more precise radiation risk estimates of solid cancer as cohort members are approaching their 60s and cancer becomes more common.

## Introduction

The high proliferative and differential potential of embryonic and fetal cells and tissues is suggestive of elevated carcinogenic radiosensitivity of the prenatal organism. Current evidence shows that X-ray irradiation of women during pregnancy leads to an increased risk of cancer in their offspring during childhood [1, 2]. Increased risk of adult-onset solid cancers is also observed in atomic bomb survivors exposed in utero [3]. However, the magnitude of risk requires further quantification. The main limitation of previous studies is inadequate statistical power because of the small sample sizes and consequently the small number of observed cancer cases [4]. Moreover, it is unknown at present whether chronic exposure at low dose rates, including that caused by the ingress of radionuclides into the bodies of pregnant women, can influence the risk of solid cancers in their offspring.

In the framework of the project “Epidemiological Studies of Exposed Southern Urals Populations (SOLO)”, analysis of cancer risk was performed in a unique combined cohort of people who were exposed in utero to elevated levels of ionizing radiation in the Southern Urals region. The operation of the Mayak Production Association (Mayak PA), a large nuclear facility in the Southern Urals, in the 1950s resulted in chronic exposure of large groups of workers and residents [5]. The internal exposure of the offspring of the Mayak PA female workers was caused predominantly by external γ-radiation, and also by intake of ^239^Pu into the bodies of pregnant women. In utero and postnatal exposure of the offspring also occurred due to gas-aerosol emissions from the Mayak PA into the atmosphere and radiation accident in 1957. A proportion of in utero exposed offspring were later exposed to radiation during their professional activities at the Mayak PA. The exposure of pregnant women living in the Techa riverside villages was caused by an increased γ-radiation background in the riverside territories, in the populated areas, and in dwellings, and also by intake of uranium fission products (first, the long-lived radionuclides ^137^Cs and ^90^Sr) with water and locally produced food. After birth, the exposure of the offspring continued due to both external γ-radiation and intake of ^89,90^Sr and other radionuclides into the child’s body with the mother’s breast milk and home-produced food and river water.

In spite of the diverse nature of the internal exposure received by the cohort members, analysis of the completeness and quality of the epidemiological and dosimetric data enabled us to pool the two cohorts (in utero exposed offspring of Mayak PA female workers and of Techa riverside female residents) to perform a joint risk analysis in the UPEC. The two cohorts were created with similar aims, under similar constraints for follow-up, and were very close to each other geographically and chronologically. The medical follow-up of the health status for in utero exposed members of both cohorts was conducted regularly [6, 7]. To ensure adequate follow-up of the cohort members, cancer registries and cause-of-death registries were created [8, 9] based on the same types of information sources on vital status, cancer cases, and causes of death. With the aim of achieving unification of the coding procedures, an inter-institutional comparison of coding of causes of death was performed [10].

Previously, no clear effects due to in utero exposure were observed based on data on solid cancer mortality in the offspring of Mayak PA female workers [11]. The UPEC members have now reached an age at which cancer becomes more common (they are almost 60 years old), and therefore an updated cancer risk analysis was of particular interest. The objective of this study was to investigate solid cancer risk after in utero irradiation in a pooled cohort of subjects born to Mayak PA female workers and Techa riverside female residents (the exposure received by UPEC members covered the total, or almost the total, gestation period).

## Methods

### Study Cohort

The UPEC was created by pooling of the two cohorts, and includes all individuals born alive in 1950–1961 to mothers who either during their pregnancy lived in the contaminated Techa riverside areas or were at any time members of the Mayak workers cohort. The Techa River In Utero Exposed Cohort (TRCIU) consists of offspring born to women who were permanent residents of the Techa riverside villages. The criteria of the inclusion into the UPEC cohort were:

- residence of mothers in one of the 41 Techa riverside villages at any time from 1 January 1950 to 31 December 1960.
- birth of alive offspring at any time over the period from 2 January 1950 to 30 September 1961 after the exposure of mothers The Mayak Workers’ Offspring Cohort (MWOC) includes all children born between January 1948 and December 1988 to women who are members of the Mayak Workers Cohort [12] (i.e. women who started employment at either nuclear reactor plant, the radiochemical plant, plutonium production plant, water treatment, or mechanical repair plants of Mayak PA in 1948–1982). The UPEC included a subset of MWOC members who were born alive in 1950–1961.

Age-sex characteristics of the cohorts are provided in [13, 14].

The follow-up period for the UPEC members was 1950–2009 (60 years). The only exception was the follow-up period for cancer incidence in offspring of the Techa riverside female residents, which was 1956–2009, since the systematic registration of cancer cases at the Chelyabinsk and Kurgan oblast oncology dispensaries began only in 1956. The catchment area for the residents of the Techa riverside villages for analysis of cancer incidence included the city of Chelyabinsk, the city of Ozyorsk, five raions contaminated with radionuclides in the Chelyabinsk oblast (Krasnoarmeisky, Kunashaksky, Kaslinsky, Argayashsky, and Sosnovsky raions) and two raions in the Kurgan oblast (Kataisky and Dalmatovsky raions). For cancer mortality analysis, the territory comprising the Chelyabinsk oblast and the Kurgan oblast was used. The catchment area for the offspring of the Mayak PA female workers for the analysis of both incidence and mortality included the city of Ozyorsk. In order to allow comparability of the members of the two cohorts in terms of age and risk period, the unified UPEC cohort comprises only offspring born in 1950–1961, given the strong secular time trends of most major solid cancer types. The number of cohort members was 16,821 (11,490 TRCIU and 5,331 MWOC members) for mortality analyses and 15,813 (10,482 TRCIU and 5,331 MWOC members) for incidence analyses (Table 1). As can be seen from Table 1, at present about half of the cohort members are still under observation (alive and living in the catchment area), more than a quarter of the cohort members have migrated out of the catchment areas, and 13% and 18% in the cohorts for analyses of cancer incidence and mortality, respectively, have died in their catchment area, with the cause of death known for about 90% of the deceased. The proportion of people lost to follow-up was 34% for mortality analyses and 39% for incidence analyses, due to the high proportions of migration (27% and 33%, respectively). The ratio of men to women in the cohort was about 1:1. Among the Techa riverside residents, there were more Slavs than Tatars and Bashkirs; the ethnicity of the offspring of the Mayak PA female workers (residents of the city of Ozyorsk) was not recorded. Mean attained age of the pooled alive cohort members in the incidence study at the end of follow up was 53 (with a range from 48 to 59, and median of 53 and interquartile range from 50 to 56.

**Table 1:** Characterization of the status at the end of the follow up of the Urals Prenatally Exposed Cohort:, sex, birth year, ethnicity, and dose.

Characteristic		Number (%)														
		Mortality							Incidence							
		No. observed	Alive	Died from solid cancer	Died from other causes	Lost to follow-up		Person-years	No. observed	Alive	Died	Solid cancer diagnosis*	Lost to follow-up			Person-years
						Status unknown	Migrants						Other cancer cases**	Status unknown	Migrants	
		16,821 (100)	7,954 (47.3)	196 (1.2)	2903 (17.3)	1,230 (7.3)	4,538 (27.0)	601,372	15,813 (100)	7,093 (44.9)	2,130 (13.5)	369 (2.3)	56 (0.4)	885 (5.6)	5,280 (33.4)	554,411
Sex	Male	8,552 (50.8)	3,597	115	1,970	653	2217	300,159	8,024 (50.7)	3,262	1,521	175	31	482	2,553	278,324
	Female	8,269 (49.2)	4,357	81	933	577	2321	301,213	7,789 (49.3)	3,831	609	194	25	403	2,727	276,087
Birth year	1950–1953	5,704 (33.9)	2,244	92	1,239	316	1813	195,336	4,998 (31.6)	1,996	680	168	19	154	1,981	174,059
	1954–1957	6,106 (36.3)	2,916	69	1,022	480	1619	221,090	5,860 (37.1)	2,569	836	128	25	360	1,942	205,566
	1958–1961	5,011 (29.8)	2,794	35	642	434	1106	184,946	4,955 (31.3)	2,528	614	73	12	372	1,356	174,786
Ethnicity/place of residence	Slavs	7,607 (45.2)	3,503	92	1,355	780	1877	273,442	6,907 (43.7)	2,899	860	158	19	527	2,444	236,578
	Tatars and Bashkirs	3,883 (23.1)	2,266	38	874	347	358	154,537	3,575 (22.6)	2,081	626	80	7	255	526	145,371
	Subset of MWOC	5,331 (31.7)	2,185	66	674	103	2303	173,393	5,331 (33.7)	2,113	644	131	30	103	2,310	172,462
Dose	< 1 mGy	9969 (59.3)	4888 (61.5)	105 (53.6)	1579 (54.4)	817 (66.4)	2580 (56.9)	362488	9592 (60.7)	4358	1301	195	31	644	1062	336538
	1–4 mGy	2580 (15.3)	1316 (16.5)	36 (18.4)	537 (18.5)	179 (14.6)	512 (11.3)	97706.9	2323 (14.7)	1150	332	71	3	115	652	87921.9
	5–19 mGy	2054 (12.2)	931 (11.7)	29 (14.8)	427 (14.7)	146 (11.9)	521 (11.5)	73323	1809 (11.4)	828	244	56	7	75	599	66064.6
	20–79 mGy	1418 (8.4)	564 (7.1)	19 (9.7)	251 (8.6)	68 (5.5)	516 (11.4)	45950.9	1304 (8.2)	518	159	33	10	36	548	42674.9
	80+ mGy	800 (4.8)	255 (3.2)	7 (3.6)	109 (3.8)	20 (1.6)	409 (9.0)	21903.4	785 (5.0)	239	94	14	5	15	418	21166.8

*ICD-9 Codes 140–199 excluding 173

**Other cancers: hematological malignancies (ICD-9 Codes 200–208), non-melanoma skin cancers (ICD-9 Code 173) and unspecified tumours (ICD-9 Code 239)

### Exposure Assessment

To study the solid cancer risk, the soft tissue doses were estimated for each UPEC member. The characteristics of dose distributions for the TRCIU and MWOC are given in Table 2.

**Table 2:** Characteristics of dose distributions for the mortality analysis of the Techa River *in Utero* Exposed Cohort (TRCIU; *n* = 11,490), the Mayak Female Workers’ Offspring Cohort exposed in utero (MWOC; *n* = 5,331), and Urals Prenatally Exposed Cohort (UPEC, n = 16,821).

Cohort	Period of exposure	Absorbed dose, mGy				
		Mean	25th percentile	Median	75th percentile	Maximum
TRCIU	In utero	4.4	0.04	0.3	2.2	294.5
	Postnatal	10.4	0.1	1.3	8.6	397.1
MWOC	In utero	35.0	0*	0	30.4	944.9
	Postnatal	12.8	0	0	0	552.0
UPEC	In utero	14.1	0	0.3	5.2	944.9
	Postnatal	11.2	0	0.4	5.3	552.0

For TRCIU members, individual doses (both in utero and postnatal) were estimated using the Techa River Dosimetry System 2009D (TRDS-2009D) [15], which includes an algorithm for in utero dose calculations. Evaluations of soft tissue doses were based on average external dose rates in specific residential areas and village-average intake functions. The dose individualization took into account age, sex, and individual residential history in the contaminated areas, in the Techa riverside villages and/or in areas of the East Urals Radioactive Trace contaminated through fallout from the nuclear accident that happened at the Mayak PA in 1957 [15]. ^89,90^Sr and ^137^Cs accumulated in the fetus were assumed as the sources of internal in utero exposure. The fetal biokinetic and dosimetric models for Sr adapted for the Techa River population were used for dose calculation [16–19]. ICRP-88 models [20] were applied to calculate the in utero doses from ^137^Cs. Dietary intakes of ^89,90^Sr and ^137^Cs for adult Techa riverside residents [21, 22] were adjusted for the increase in food consumption by pregnant women [23]. External in utero doses were calculated using TRDS-2009D, considering exposure of the maternal uterus as a surrogate of the fetal exposure. Postnatal doses were also calculated based on TRDS-2009D, using the individual data on residence in specific Techa riverside villages. The doses due to intakes of short-lived radionuclides were considered. Breast milk as a source of ^89,90^Sr and ^137^Cs for infants was taken into account [17]. In current cancer risk analysis, the stomach dose resulting from postnatal exposure was used as a proxy of the postnatal dose to soft tissues, because of the high prevalence of stomach cancer and also the fact that the ratio of the dose accumulated in most organs to the stomach dose is close to 1. As can be seen from Table 2, the dose distributions are characterized by large variations and do not follow normal or log-normal distributions. Postnatal doses to soft tissues were markedly higher than in utero ones. External and internal exposure to ^137^Cs contributes about 80% of the total soft tissue dose.

Imperfect technologies and radiation safety standards resulted in higher occupational doses of external exposure in Mayak PA female workers who started working at the facility in the earlier years of its operation (1948–1958). In the current study, only doses of external γ-radiation exposure were taken into account, which were based on the measurements of the film badge dosimeters used for individual dosimetry monitoring. 2754 members (52%) of the MWOC had an estimated zero in-utero radiation dose.A proportion of MWOC members (1,450 people) were exposed postnatally, after they started working at the Mayak PA. For 889 of those individuals, the postnatal doses of γ-radiation were reconstructed according to the Mayak Worker Dosimetry System 2008 [24].

### Data Collection

The methods of follow-up of the members of both cohorts were developed and consolidated by experience many years ago [11, 25]. The vital status of the cohort members was regularly assessed on the basis of the data derived from the Chelyabinsk and Kurgan oblasts’ address bureaus, the Mayak Unified Computer Registry, and the results of interviews with relatives; the address bureaus were the most accurate source.

For incidence analyses, data from the cancer registry were used, and the mortality analyses were based on data from cause-of-death registries. The basic sources of information on cancer cases included medical records (notifications of the first diagnosed cancer cases, patient medical records, operation log books, results of analyses performed by the cytology and pathology laboratories, etc.) obtained from health care providers such as the Chelyabinsk and Kurgan oblast oncology dispensaries and Central Medical Sanitary Department 71, and the information on cancer mortality was derived from death registration certificates and medical death certificates. To verify the causes of death, the pathology protocols of the specialized medical centers (oblast oncology dispensaries, Central Medical Sanitary Department 71, oblast clinics, etc.) were used. The causes of death were coded based on the International Classification of Diseases, Ninth Revision (ICD-9) by trained nosologists and entered into the cause-of-death registries.

The prerequisites for the combined analysis of cancer in the unified cohort of prenatally exposed people in the Southern Urals region were the narrow age range, similar time period for following up the cohort members, the comparable methodology of the follow-up within the framework of which analogous information sources were used for the vital status as well as cancer incidence and mortality, residence in the same geographical area of the Russian Federation (the Chelyabinsk and Kurgan oblasts), and estimated individual doses of in utero and postnatal exposure. It should be noted, however, that the socioeconomic conditions differed between the members of the two cohorts. The MWOC members were city residents and had better living conditions than the TRCIU members, who were rural residents of the Techa riverside villages.

Overall, during the follow-up period of up to 60 years, 369 incident cases of solid cancer (excluding 6 cases of non-melanoma skin cancers, ICD-9 Code 173) and 196 deaths from cancer were registered in the UPEC. In children younger than 15 years, only 10 cases and 8 deaths from solid cancers were registered; among those 10 incident cases 4 were observed in the MWOC and 6 in the TRCIU, and 2 in MWOC and 6 in TRCIU for the 8 who died respectively. The most common cancers overall were cancers of the digestive system and respiratory system and breast cancer (Table 3).

**Table 3:** Distribution of incident cancer cases in the UPEC.

		Men			Women	
Site (ICD-9 Code)	N	%	Mean age(Range)	N	%	Mean age(Range)
DigestiveOesophagus (150)Stomach (151)Colorectal (153–154)Liver (155)Other digestive (152,156–159)	4921816211		45(1–58)48 (47–49)45(29–56)47(34–58)41(40–42)41(1–58)	430142126		47(11–59)-48(40–58)47(25–5929 (11–47)47(37–56)
RespiratoryLung (162)Other respiratory (160, 161, 163–165)	584810		48(27–59)49(27–59)46(29–54)	651		43(37–51)44(37–51)40
Breast (174–175)	0			49		47(31–58)
Other sites (same as last line?)Cervix (180)Prostate (185)Bladder (188)Brain/CNS (191–192)Thyroid (193)Other (within 140–199)	-0211649		35(29–41)31(8–52)39(33–44)41(1–57)	20-061951		41 (27–56)—42(29–50)43(24–54)41(1–58)

Excluding non-melanoma skin cancer (173).

### Ethics Statement

This record-based epidemiological study did not require any contact with the cohort members. Information was anonymized and de-identified prior to analysis. The study was approved by the Ethics committee of the Urals Research Center for Radiation Medicine (URCRM), Chelyabinsk, Russia.

### Statistical Analysis

We used Poisson regression methods to quantify the relative risk (RR) as a function of the in utero soft tissue dose and the postnatal stomach dose for solid cancer incidence and mortality separately. The person-year table was stratified on ethnicity/place of residence (Slavs, Tatars and Bashkirs, Ozyorsk residents), sex, 5-year categories of attained age and of calendar year, birth period (1950–1953, 1954–1957, 1958–1961), and on 10 mGy intervals of in utero and postnatal doses. The analyses were based on linear RR models of the form *λ*_0_(*a*,*s*,*r*)exp[*βd*_*iu*_ + *δd*_*pn*_], where *λ*_0_(.) is the baseline hazard rate function, modelled as a function of log(age/45), sex (*s*), and place of residence (*r*). Hypothesis tests and confidence intervals were based on likelihood ratio tests and direct evaluation of the profile likelihood. Data management was performed in Stata [26] and risk analyses in Epicure [27].

To analyze the dose–effect dependence, in cases of in utero exposure the doses to soft tissues were used, and in cases of postnatal exposure the doses to the stomach were used. No lag period was taken into account for estimation of the effect of the in utero dose, whereas for the postnatal dose the lag was taken as 1 year (for risk of childhood cancer) and 5 years (for cancer risk in adults).

## Results

Table A in S1 File shows the RRs for solid cancer incidence and mortality related to in utero exposure, including the person-years at risk and the number of observed cases. When a linear model was used, no increase in the risk of solid cancer was seen based on incidence data or on mortality data. In categorical analyses, the highest RRs were observed in the second lowest exposure category (1–4 mGy) and the lowest RRs in the highest exposure category (> 80 mGy).

The analysis of incidence by cancer site did not show any consistent relationships with dose for cancers of the digestive system or the respiratory system, or for breast cancer in women (Table B in S1 File). A statistically significantly increased RR of incidence was noted for cancer of the respiratory system in the second lowest exposure category (1–4 mGy).

Taking into consideration that many cohort members were exposed not only in utero but also postnatally, analyses of RRs for cancer incidence and mortality were performed by modelling both exposure periods simultaneously. As can be seen from Table 4, the RRs for cancer incidence and mortality showed a tendency towards a decrease with increasing in utero doses, and the lowest RRs were observed for the highest in utero doses. In contrast, a positive relationship was observed between cancer risk and postnatal exposure. In the highest exposure category (> 80 mGy), the increase in the RR of cancer incidence was statistically significant.

**Table 4:** Relative risks for solid cancer incidence and mortality with mutual adjustment for *in utero* and postnatal exposure to radiation in the Urals Prenatally Exposed Cohort.

Dose	Incidence*			Mortality		
	Person-years	No. observed	RR (95% CI)	Person-years	No. observed	RR (95% CI)
**In utero dose, mGy**						
< 1	336,583	195	(Referent)	362,488	105	(Referent)
1–4	87,922	71	1.32 (0.97 to 1.77)	97,707	36	1.21(0.79 to 1.82)
5–19	66,065	56	1.19 (0.86 to 1.62)	73,323	29	1.14 (0.72 to 1.74)
20–79	42,675	33	0.97 (0.64 to 1.41)	45,951	19	1.08 (0.63 to 1.78)
>80	21,167	14	0.72 (0.39 to 1.22)	21,903	7	0.65 (0.26 to 1.37)
**Postnatal dose, mGy**						
< 1	325,580	161	(Referent)	351,742	83	(Referent)
1–4	88,475	59	0.90 (0.64 to 1.27)	98,329	36	0.98 (0.62 to 1.53)
5–19	81,880	65	1.02 (0.73 to 1.43)	88,428	33	0.92 (0.57 to 1.45)
20–79	44,061	53	1.17 (0.82 to 1.66)	47,355	28	1.07 (0.65 to 1.71)
>80	14,416	31	1.72 (1.12 to 2.57)	15,519	17	1.56 (0.87 to 2.67)
**Linear model of the doses**						
Linear/10 mGy in utero	554,411	369	0.98 (0.96 to 1.01)	601,372	196	0.98 (0.94 to 1.01)
Linear/10 mGy postnatal	554,411	369	1.02 (1.00 to 1.04)	601,372	196	1.02 (0.99 to 1.05)

*non-melanoma skin cancers excluded

Table 5 shows the RRs for incidence for different cancer sites with in utero exposure and postnatal exposure taken into account simultaneously. For cancers of the digestive system, no dependence of RR on in utero dose was observed, whereas a statistically significant increase in RR with increasing postnatal dose to the stomach was seen; the increase was statistically significant only in the highest exposure category (> 80 mGy).

**Table 5:** Relative risks for cancer incidence in the digestive system, respiratory system, and breast (women only) with mutual adjustment of *in utero* and postnatal exposure in the Urals Prenatally Exposed Cohort.

Dose	Digestive cancers (ICD-9 codes 150–159)*			Respiratory cancers (ICD-9 codes 160–165)*			Breast cancer (ICD-9 code 174)**		
	Person-years	No. observed	RR (95% CI)	Person-years	No. observed	RR (95% CI)	Person-years	No. observed	RR (95% CI)
**In utero dose, mGy**									
< 1	336,583	42	(Referent)	336,583	29	(Referent)	165,838	32	(Referent)
1–4	87,922	17	1.38 (0.74 to 2.51)	87,922	20	2.02 (1.07 to 3.76)	45,920	10	1.16 (0.51 to 2.45)
5–19	66,065	16	1.53 (0.80 to 2.78)	66,065	10	1.26 (0.57 to 2.61)	32,540	3	0.45 (0.11 to 1.31)
20–79	42,675	13	1.72 (0.85 to 3.30)	42,675	2	0.40 (0.06 to 1.39)	21,489	3	0.76 (0.18 to 2.21)
>80	21,167	3	1.05 (0.31 to 2.72)	21,167	3	1.38 (0.32 to 4.26)	10,300	1	0.41 (0.02 to 1.96)
**Postnatal dose, mGy**									
< 1	325,580	34	(Referent)	325,580	16	(Referent)	166,291	27	(Referent)
1–4	88,475	14	1.02 (0.51 to 1.94)	88,475	15	1.40 (0.64 to 3.03)	44,819	9	0.78 (0.33 to 1.65)
5–19	81,880	15	0.97 (0.49 to 1.84)	81,880	13	1.11 (0.49 to 2.48)	40,576	8	0.73 (0.29 to 1.66)
20–79	44,061	17	1.26 (0.65 to 2.37)	44,061	12	1.63 (0.70 to 3.67)	19,086	4	0.50 (0.14 to 1.36)
>80	14,416	11	2.29 (1.09 to 4.56)	14,416	8	2.62 (1.02 to 6.26)	5,315	1	0.29 (0.02 to 1.41)
**Linear model of the doses**									
Linear/10 mGy in utero	554,411	92	1.01 (0.97 to 1.04)	554,411	64	1.00 (0.92 to 1.05)	276,087	49	0.90 (0.71 to 1.01)
Linear/10 mGy postnatal	554,411	92	1.04 (1.00 to 1.07)	554,411	64	1.03 (0.98 to 1.06)	276,087	49	0.95 (0.82 to 1.03)

* adjustment on sex, age, and ethnicity/place of residence

** adjustment on age (linear and quadratic terms), among women only

For cancers of the respiratory system, the dependence of RRs for incidence on in utero and postnatal dose was similar to that for cancers of the digestive system. A statistically significant increase in RR was seen for the second lowest in utero exposure category (1–4 mGy) and was also observed for the highest postnatal exposure category (> 80 mGy). For breast cancer risk in women, the results differed from those for the respiratory and digestive system: the RRs decreased not only with increasing in utero dose but also with increasing postnatal dose, although none of the RR estimates were statistically significant.

Taking into consideration the observation that the doses of in utero and postnatal exposures were correlated (*r* = 0.37) in the offspring of the Techa riverside residents, in order to minimize the influence of the postnatal exposure on the risk of cancer development in people exposed in utero, cancer incidence analysis was performed for the UPEC subcohort truncated by cutting off the postnatal dose at 10 mGy. Among 14,519 members of the truncated cohort whose postnatal stomach dose did not exceed 10 mGy, 241 cases of cancer occurred. The linear model confirmed the earlier observed tendency towards a decreased risk of all solid cancers with increasing in utero dose (Table 6), although this was not statistically significant. Similarly, no associations were seen with any of the cancer sites investigated separately (data on the respiratory system are not shown in the table).

**Table 6:** Relative risks for incidence of all solid cancers, cancers of the digestive system and of the breast (women only), in a subcohort truncated at the postnatal dose of 10 mGy.

In utero dose, mGy	All solid cancers (ICD-9 codes 140–199, except code 173 –non-melanoma skin cancer)*			Digestive cancers (ICD-9 codes 150–159)*			Breast cancer (ICD-9 code 174)**		
	Person-years	No. observed	RR (95% CI)	Person-years	No. observed	RR (95% CI)	Person-years	No. observed	RR (95% CI)
< 1	303,271	150	(Referent)	303,271	28	(Referent)	152,733	25	(Referent)
1–4	60,573	40	1.39 (0.96 to 1.97)	60,573	10	1.70 (0.78 to 3.46)	31,935	8	1.45 (0.61 to 3.09)
5–19	42,325	28	1.18 (0.77 to 1.73)	42,325	7	1.54 (0.62 to 3.35)	20,671	2	0.50 (0.08 to 1.70)
20–79	24,143	13	0.89 (0.47 to 1.54)	24,143	3	1.27 (0.29 to 3.93)	12,666	2	0.90 (0.14 to 3.02)
>80	17,945	10	0.79 (0.38 to 1.47)	17,945	4	1.95 (0.54 to 5.63)	9,349	1	0.56 (0.03 to 2.65)
Linear/10 mGy	448,256	241	0.98 (0.95 to 1.01)	448,256	52	1.02 (0.97 to 1.06)	227,354	38	0.94 (0.76 to 1.03)

*adjusted for sex, age (linear and quadratic coefficient), and ethnicity/place of residence (Slavs, Tatars and Bashkirs, Ozyorsk residents)

**adjusted for age (linear and quadratic coefficient) among women only

## Discussion

This paper examines both childhood and adult cancer risk associated with chronic exposure to ionizing radiation of the embryo and fetus. The radiation exposure covered the total gestation period or a substantial portion of it. Prenatal exposure of different organs caused by both external and internal exposure (^89,90^Sr and ^137^Cs) allowed for analyses of solid cancers at different organ sites. Individual estimates of doses to soft tissues of the embryo and fetus as well as postnatal doses to the stomach made it possible to analyze the dose dependence of cancer incidence and mortality over a wide range of doses. The results did not show increased risks of chronic in utero low-dose-rate exposure with doses up to 944.9 mGy. In contrast, a tendency towards a decrease in RR was seen with increasing in utero doses to soft tissues, although not statistically significant.

In comparison with our findings, the atomic bomb survivors exposed in utero–a smaller cohort of 2,452 subjects–showed an excess relative risk of 1.0 (95% CI 0.2–2.3) per Sv based on 94 cancers occurring before the age of 50 [3]. Reasons for the discrepancy may be lower doses in our study or lower dose rate, or differences in the baseline cancer risks affecting the detection of radiation-related excess risks, as well as methodological issues such as uncertainty in dose estimation or effects from losses to follow up, or simply a play of chance as numbers for cancer sub-types are small in both studies.

Our observed indication of a trend of decreasing risk with increasing exposure can perhaps be explained by the induction of lethal genetic changes with higher doses of in utero exposure in the cells of the embryo and the fetus, which are capable of high proliferative activity, and subsequent elimination of the compromised offspring during the in utero and early postnatal periods. In earlier studies of female residents of Techa riverside villages who were exposed during pregnancy, no strong evidence of increases in prenatal loss (spontaneous abortions, miscarriages) or impairment of gestation was found [6]. However, it was shown that the infant mortality rate significantly depended on fetal dose and dose to red bone marrow received during the first year of life, with a 3% increase in risk per 10 mGy of in utero and postnatal doses [28]. Although the data from animal studies on radiation-induced embryonic or fetal death appears rather inconsistent, increased lethality was observed in studies of mice after exposures in the dose range 0.05–0.5 Gy on day 7 after conception [29, 30].

To the contrary, positive dependence of solid cancer incidence RR on postnatal dose was observed in UPEC. In the highest dose category (> 80 mGy) RR was statistically significantly increased. Pooling the datasets of the two cohorts (TRCIU and MWOC) resulted in substantially higher statistical power for both cancer mortality and cancer morbidity. The strengths of the study are the large sample size, long-term follow-up, and individualized dose estimates. However, the study also has some limitations.

As already mentioned, members of the cohort were exposed not only in utero but also during the postnatal period, due to their residence in the Techa riverside villages or in areas of the East Urals Radioactive Trace, or due to their professional activities at the Mayak PA. Postnatal doses to the stomach often exceeded in utero soft tissues doses, and reached 552.0 mGy. The interdependence of in utero and postnatal doses for the offspring of female residents of Techa riverside villages limited the attempts to completely eliminate the influence of the postnatal exposure on the in utero results.

The reproductive organs of the parents of the UPEC members were also exposed before conception (only maternal gonads, or both maternal and paternal gonads if the fathers were resident in contaminated villages or worked at the Mayak PA). The effects of the pre-conception exposure of the parental gonads on the risk of cancer in people with prenatal exposure are not considered in this study. However, an earlier study in the Techa River offspring cohort provided no evidence for an association between the pre-conception exposure of the parents and the risk of cancer in their offspring [31]. Another limitation of the study is the incomplete information on potential confounding factors, for example the ethnicity of the offspring of the Mayak PA female workers, and the smoking habits or other major non-radiation cancer risk factors of the cohort members. Exposure to other radiation sources, in particular from medical sources, was also not known.

Uncertainty in risk assessments is associated with the uncertainty of epidemiological data, risk models and dose estimates. The uncertainties of dose estimates for the Techa riverside residents resulted from many factors [31, 32], including the use of village-average intakes of radionuclides or average external dose rates in residential areas. However, dosimetry data that were used for the evaluation of the external and internal doses include numerous radionuclide measurements in Techa riverside residents and measurements of γ-radiation fields in residential areas and on Techa riverbanks. The stochastic version of TRDS-2009 [33] enables the individual dose uncertainty to be quantified, which was done for Techa River Cohort members. It was shown that uncertainty ranges are variable and depend upon how much individual-specific information was available. For well-documented people, individual doses appear to be log-normally distributed with geometric standard deviations of about 2–2.5; for people with less information available, uncertainties are larger, with geometric standard deviations of 3 or more.

As noted earlier, the doses of external γ-radiation exposure for Mayak PA female workers were based on adjusted film badge readings and group monitoring, therefore the uncertainties of doses to the embryo and fetus of these women still remained. The dose estimates for the MWOC did not take into account the contributions of ^90^Sr and ^137^Cs (mainly resulting from the nuclear accident in 1957), and ^131^I (from Mayak PA airborne releases) to the exposures of mothers and their children due to residence in the city of Ozyorsk. Contributions from ^239^Pu (received by mothers during their professional activities) were not considered in this analysis although available for a subset, but doses were low and no association was observed with solid cancer risk [14].

Cancer incidence is strongly related to age and reaches a peak at age 60–75 years [34, 35]. UPEC members have now attained the age of 60 years. Therefore, due to the still small number of cancer cases for analyses, it was difficult to estimate the risk of site-specific cancer, or even to obtain reliable estimates of radiation-related risk of solid cancers for the whole pooled cohort. If follow-up were continued for a further 10 years, the number of cancer cases in the cohort, and thus the statistical power of the study, would increase substantially, as about half of the cohort is still alive and living in the catchment area. Consequently, the UPEC remains a cohort that is unique worldwide for analyses of effects of chronic in utero radiation exposure, and that merits further research.

## Supporting Information

S1 File Table A. Relative risks for solid cancer incidence and mortality in different groups of in utero dose in the Urals Prenatally Exposed Cohort. Table B. Relative risks for solid cancer incidence in the digestive system, respiratory system, and breast (women only) in the Urals Prenatally Exposed Cohort.
(DOCX)

## Abbreviations

CI: confidence interval
ICD-9: International Classification of Diseases, Ninth Revision
Mayak PA: Mayak Production Association
MWOC: Mayak Workers’ Offspring Cohort
RR: relative risk
SOLO Project: Epidemiological Studies of Exposed Southern Urals Populations
SOUL Project: Southern Urals Radiation Risk Research
TRDS-2009D: Techa River Dosimetry System 2009D
TRCIU: Techa River In Utero Exposed Cohort
UPEC: Urals Prenatally Exposed Cohort
