# Supplementary material for: Incidence and Mortality of Solid Cancers in People Exposed In Utero to Ionizing Radiation: Pooled Analyses of Two Cohorts from the Southern Urals, Russia
Source: PLoS One. 2016 Aug 3;11(8):e0160372. doi: 10.1371/journal.pone.0160372 (PMC4972315; doi:10.1371/journal.pone.0160372)
Supplement: S1 File — Table A. Relative risks for solid cancer incidence and mortality in different groups of in utero dose in the Urals Prenatally Exposed Cohort. Table B. Relative risks for solid cancer incidence in the digestive system, respiratory system, and breast (women only) in the Urals Prenatally Exposed Cohort. (DOCX) [file pone.0160372.s001.docx]

**Table A. Relative risks for solid cancer incidence and mortality in different groups of in utero dose in the Urals Prenatally Exposed Cohort**

| **In utero dose, mGy** | **Incidence*** | | | **Mortality** | | |
| --- | --- | --- | --- | --- | --- | --- |
|  | **Person-years** | **Cancer cases** | **RR (95% CI)** | **Person-years** | **Cancer cases** | **RR (95% CI)** |
| < 1 | 336,583 | 195 | (Referent) | 362 488 | 105 | (Referent) |
| 1–4 | 87,922 | 71 | 1.28 (0.95 to 1.69) | 97,707 | 36 | 1.16 (0.77 to 1.72) |
| 5–19 | 66,065 | 56 | 1.20 (0.88 to 1.62) | 73,323 | 29 | 1.12 (0.72 to 1.70) |
| 20–79 | 42,675 | 33 | 1.04 (0.70 to 1.51) | 45,951 | 19 | 1.13 (0.66 to 1.83) |
| >80 | 21,167 | 14 | 0.75 (0.40 to 1.27) | 21,903 | 7 | 0.69 (0.28 to 1.44) |
| Linear/10 mGy | 554,411 | 369 | 0.99 (0.96 to 1.01) | 601,372 | 196 | 0.98 (0.94 to 1.01) |

*non-melanoma skin cancers excluded

**Table B. Relative risks for solid cancer incidence in the digestive system, respiratory system, and breast (women only) in the Urals Prenatally Exposed Cohort**

| **In utero dose,**  **mGy** | **Digestive cancers (ICD-9 codes 150-159)** | | | **Respiratory cancers (ICD-9 codes 160-165)** | | | **Breast cancer (ICD-9 code 174)** | | |
| --- | --- | --- | --- | --- | --- | --- | --- | --- | --- |
|  | **Person-years** | **No. observed** | **RR (95% CI)** | **Person-years** | **No. observed** | **RR (95% CI)** | **Person-years** | **No. observed** | **RR (95% CI)** |
| < 1 | 336,583 | 42 | (Referent) | 336,583 | 29 | (Referent) | 165,838 | 32 | (Referent) |
| 1–4 | 87,922 | 17 | 1.32 (0.72 to 2.31) | 87,922 | 20 | 2.07 (1.13 to 3.71) | 45,920 | 10 | 1.00 (0.17 to 1.97) |
| 5–19 | 66,065 | 16 | 1.54 (0.82 to 2.75) | 66,065 | 10 | 1.35 (0.62 to 2.70) | 32,540 | 3 | 0.38 (0.09 to 1.06) |
| 20–79 | 42,675 | 13 | 1.97 (0.99 to 3.66) | 42,675 | 2 | 0.49 (0.01 to 1.65) | 21,489 | 3 | 0.61 (0.15 to 1.70) |
| >80 | 16,991 | 3 | 1.15 (0.34 to 2.94) | 16,991 | 3 | 1.62 (0.37 to 5.03) | 10,300 | 1 | 0.40 (0.02 to 1.85) |
| Linear/10 mGy | 554,411 | 92 | 1.01 (0.97 to 1.04) | 554,411 | 64 | 1.02 (0.92 to 1.05) | 276,087 | 49 | 0.89 (0.70 to 1.01) |
